# Health-related quality of life with daratumumab, bortezomib, melphalan, and prednisone versus bortezomib, melphalan, and prednisone alone in transplant-ineligible patients with newly diagnosed multiple myeloma: analysis of the phase 3 OCTANS study

**DOI:** 10.1007/s00277-025-06303-3

**Published:** 2025-05-23

**Authors:** Weijun Fu, Soo-Mee Bang, Honghui Huang, Kihyun Kim, Wei Li, Gang An, Je-Jung Lee, Zhen Cai, Jie Jin, Yafei Wang, Chor Sang Chim, Robin Carson, Rui Liu, Man Zhao, Xi Chen, Canchan Cui, Jian Hou, Jianxiang Wang

**Affiliations:** 1 Shanghai Changzheng Hospital, Shanghai, China; 2 Seoul National University Bundang Hospital, Seongnam, South Korea; 3 Renji Hospital, Shanghai Jiao Tong University School of Medicine, Shanghai, China; 4 Samsung Medical Center, Sungkyunkwan University School of Medicine, Seoul, South Korea; 5 First Hospital of Jilin University, Changchun, China; 6 Chinese Academy of Medical Sciences & Peking Union Medical College, Tianjin, China; 7 Chonnam National University Medical School, Hwasun, Jeollanamdo South Korea; 8 First Affiliated Hospital of Zhejiang University, College of Medicine, Hangzhou, Zhejiang China; 9 Tianjin Cancer Hospital, Tianjin, China; 10 Hong Kong Sanatorium & Hospital, Happy Valley, Hong Kong; 11 Johnson & Johnson, Spring House, PA USA; 12 Johnson & Johnson, Beijing, China; 13 IQVIA, Shanghai, China; 14 Johnson & Johnson, Shanghai, China

**Keywords:** Daratumumab, Multiple myeloma, Transplant-ineligible, Patient-reported outcomes

## Abstract

In the phase 3 OCTANS study, daratumumab plus bortezomib/melphalan/prednisone (D-VMP) significantly improved response rates and progression-free survival versus VMP alone in transplant-ineligible Asian patients with newly diagnosed multiple myeloma (NDMM). Understanding the impact of treatment on patient-reported outcomes (PROs) is of increasing interest. Here, we report on the impact of D-VMP and VMP on PROs in OCTANS. PROs were a secondary endpoint and were assessed using the European Organisation for Research and Treatment of Cancer Quality of Life Questionnaire Core 30-item (EORTC QLQ-C30) and the EuroQol 5-dimensional descriptive system (EQ-5D-5L) questionnaire, administered at screening, every 3 months (Year 1), every 6 months thereafter (until disease progression), and 8 and 16 weeks post disease progression. Treatment effects were estimated using a mixed-effects model with repeated measures. Overall, 220 patients were randomized (D-VMP, *n* = 146; VMP, *n* = 74). Compliance rates were 100% at baseline in both treatment groups and remained relatively high by Month 12 (D-VMP, 82.6%; VMP, 67.4%). Comparable improvements from baseline were generally observed between treatment groups across most PRO scores. Significant improvements were observed with D-VMP versus VMP in Global Health Status (GHS) score at 9 months (*p* = 0.0443) and in social functioning and nausea and vomiting symptom scores at 12 months (*p* = 0.0042 and *p* = 0.0012, respectively). In summary, transplant-ineligible Asian patients with NDMM demonstrated improvements in PROs following treatment with D-VMP and VMP; however, greater improvements were observed in GHS, social functioning, and nausea and vomiting symptoms with D-VMP.

**Supplementary Information:**

The online version contains supplementary material available at 10.1007/s00277-025-06303-3.

## Trial registration

ClinicalTrials.gov Identifier: NCT03217812; submitted July 13, 2017.

## Introduction

Daratumumab is a human IgGκ monoclonal antibody targeting CD38 with direct on-tumor [1–4] and immunomodulatory [5–7] mechanisms of action. Based on the results from the primary analysis (median follow-up, 16.5 months) of the phase 3 ALCYONE study (ClinicalTrials.gov Identifier: NCT02195479), in which the addition of daratumumab to bortezomib, melphalan, and prednisone (VMP) significantly reduced the risk of disease progression or death, and increased response and minimal residual disease (MRD)–negativity rates, daratumumab in combination with VMP (D-VMP) is approved for the treatment of transplant-ineligible patients with newly diagnosed multiple myeloma (NDMM) [8].

Results from the phase 3 OCTANS study further corroborate the favorable clinical benefit of D-VMP compared with VMP alone in Asian transplant-ineligible patients with NDMM. In the primary analysis of OCTANS (median follow-up, 12.3 months), D-VMP significantly reduced the risk of disease progression or death by 57% and improved depth of response versus VMP alone [9]. In the final analysis of OCTANS, with an extended median follow-up of 41.2 months, D-VMP continued to demonstrate a significant reduction in the risk of disease progression or death (65%; *p* < 0.0001) versus VMP and further deepened responses, with a notably higher MRD-negativity (10^–5^) rate of 40.4% versus 10.8% with D-VMP and VMP, respectively, and sustained (≥ 12 months) MRD-negativity rate of 24.7% versus 1.4% [10].

While long-term outcomes have continued to improve with the rapid advancement in novel multiple myeloma (MM) treatment options, given the chronic nature of this disease, living with MM still profoundly impacts patients’ daily lives and activities of daily living [11]. The additive consequence of MM and continued treatment exposure, for example, can negatively impact physical, social, functional, and socioeconomic factors. Therefore, monitoring and maintaining health-related quality of life (HRQoL) is becoming an increasingly important clinical endpoint in the MM field. Although a few global and regional clinical studies have reported on patient-reported outcomes (PROs) of patients with NDMM receiving daratumumab-based therapies [12–14], to our knowledge, no studies have specifically addressed the impact of daratumumab-based therapies on PROs in Asian patients with NDMM, whether transplant-eligible or -ineligible, nor in Asian patients with relapsed or refractory MM. This significant gap underscores the importance of PRO data in this population.

Here, we present analyses from the OCTANS study, evaluating PROs using the European Organisation for Research and Treatment of Cancer Quality of Life Questionnaire Core 30-item (EORTC QLQ-C30) and the EuroQol 5-dimensional descriptive system (EQ-5D-5L) scales. The study compares D-VMP treatment with VMP treatment in transplant-ineligible Asian patients with NDMM.

## Methods

### Study design and patients

Full details of the methods for the phase 3, multicenter, randomized, open-label, active-controlled OCTANS study have been previously reported [9]. Patients were enrolled from 39 sites across 5 countries/regions between December 11, 2017, and December 20, 2019. Measures were taken to monitor patients during the COVID-19 pandemic to help potentially reduce missing data. Ultimately, the COVID-19 pandemic did not limit the interpretation of study results. Independent ethics committees and institutional review boards at each study site approved the study protocol and all amendments, and the study was conducted in accordance with the ethical principles outlined in the Declaration of Helsinki, International Council for Harmonisation Good Clinical Practice guidelines, and abided by all applicable regulatory requirements. Prior to participating in any study-related activities, all patients provided written informed consent.

Briefly, eligible patients were ≥ 18 years of age, had documented NDMM, were not considered a candidate for autologous stem cell transplant (due to age [≥ 65 years] or comorbidities), and had an Eastern Cooperative Oncology Group performance status score of ≤ 2. Eligible patients were randomized 2:1 to receive D-VMP or VMP treatment. All patients received up to nine 42-day cycles of bortezomib (1.3 mg/m^2^ subcutaneously twice weekly during Weeks 1, 2, 4, and 5 of Cycle 1 for a total of 8 doses per cycle, followed by once weekly during Weeks 1, 2, 4, and 5 of Cycles 2–9 for a total of 4 doses per cycle), melphalan (9 mg/m^2^ orally on Days 1–4 of each cycle), and prednisone (60 mg/m^2^ orally on Days 1–4 of each cycle). In Cycle 1, bortezomib was administered on Days 1, 4, 8, 11, 22, 25, 29, and 32. In Cycles 2–9, bortezomib was administered on Days 1, 8, 22, and 29. Those in the D-VMP group also received daratumumab (16 mg/kg intravenously once weekly in Cycle 1, once every 3 weeks in Cycles 2–9, and once every 4 weeks thereafter [Cycles ≥ 10]) until documented disease progression, unacceptable toxicity, or the end of the study.

###  PRO measures

Per protocol, the study investigators received guidance for administration of the PRO questionnaires as part of their PRO-specific training. PROs were collected using the EORTC QLQ-C30 [15] and the EQ-5D-5L [16] instruments. The EORTC QLQ-C30 is a validated, cancer-specific tool composed of 30 items that span 5 functional scales (physical functioning, role functioning, emotional functioning, cognitive functioning, and social functioning), 1 Global Health Status (GHS) scale, 3 symptom scales (fatigue, nausea and vomiting, and pain), and 6 single items (dyspnea, insomnia, appetite loss, constipation, diarrhea, and financial difficulties) [15]. Higher EORTC QLQ-C30 scores represent greater GHS, better functioning, and worse symptoms. The recall period was 1 week. The EQ-5D-5L is a generic measure of health status that assesses 5 domains, including mobility, self-care, usual activities, pain/discomfort, and anxiety/depression, plus a visual analog scale (VAS) rating for “health today” ranging from 0 (worst imaginable health state) to 100 (best imaginable health state) [16]. For the purpose of this study, the scores of the 5 domains were used to compute a single utility score (0.0–1.0). A higher utility value represents a better general health status of the patient [17]. For this analysis, pre-specified key PRO domains included the EORTC QLQ-C30 GHS and the EQ-5D-5L VAS and utility score. Secondary PRO domains included EORTC QLQ-C30 functional scales (physical, role, emotional, cognitive, and social) and symptom scales (pain, fatigue, and nausea and vomiting). Single-item scores (dyspnea, insomnia, appetite loss, constipation, diarrhea, and financial difficulties) were completed at baseline only.

Per protocol, all PRO assessments were completed at screening, every 3 months during Year 1 from baseline (Day 1, Cycle 1) onwards, every 6 months thereafter until disease progression, and at 8 and 16 weeks post disease progression. The more frequent PRO assessment schedule during Year 1 was intended to coincide with the VMP treatment period. Although treatment sessions could be rescheduled, potentially resulting in adjustments to the timing of PRO assessments, the data analysis adhered to the original calendar schedule. The rule was as follows: for month Y, the target time point was Y × 28 + 1 day. For the first year, a variation of ± 4 weeks (i.e., ± 28 days) was allowed. For the second year and beyond, a variation of ± 8 weeks (i.e., ± 56 days) was allowed. Any PRO assessment that fell outside the permitted time window was excluded from the analysis and marked as non-compliant. Assessments were completed before any other study-related procedures or evaluations using paper-based versions of the questionnaires.

In previous studies, thresholds for minimally important differences for clinically meaningful changes from baseline in PROs were established for the EORTC QLQ-C30 GHS score (≥ 8 points), the EORTC QLQ-C30 functional and symptom scores (≥ 10 points), the EQ-5D-5L VAS score (≥ 7 points), and the EQ-5D-5L utility score (≥ 0.08 points) [18, 19]. Based on these publications, in the current study, clinically meaningful changes were established as a ≥ 10-point change from baseline in EORTC QLQ-C30 scores and a ≥ 7-point change in EQ-5D-5L scores.

### Statistical analyses

The primary analysis population was the intent-to-treat (ITT) population, defined as all patients who were randomized to D-VMP or VMP. The PRO analyses included all patients in the ITT population with a baseline value, defined as the closest non-missing measurement taken on or before the first study treatment administration (Day 1, Cycle 1), and ≥ 1 post-baseline assessment. Additionally, for the EORTC QLQ-C30 specifically, 50% completion of the relevant items for a domain was required to calculate a domain score; otherwise, the domain score was recorded as missing. No imputation was done for missing data; instead, a missing-at-random approach was employed within the mixed model for repeated measures.

Compliance rates for completing the EORTC QLQ-C30 and EQ-5D-5L were determined at each time point and presented as a percentage, with the number of PRO forms received with ≥ 1 completed item on the PRO measure at the specified time point as the numerator and the number of PRO forms expected at that time point as the denominator.

Baseline PRO data were summarized descriptively, with mean and standard deviation provided. The change from baseline at each time point was also summarized descriptively for each treatment group. Change from baseline at each time point between the 2 treatments was estimated using a mixed-effects model with repeated measures, with patients as a random effect, and baseline value, treatment group, time in month, treatment-by-time interaction, and stratification factors as fixed effects. Statistical significance was tested at the 0.05 significance level. Results are presented as least squares (LS) means with 95% confidence intervals (CIs).

Results for the proportions of patients achieving minimally important differences for clinically meaningful changes from baseline in PROs are reported with exact 95% CIs.

## Results

### Patient baseline characteristics and compliance rates

A total of 220 patients were enrolled and randomized to the D-VMP group (*n* = 146) or VMP group (*n* = 74). As previously reported, patient demographics and baseline disease characteristics were generally well balanced between treatment groups (Online Resource 1); median (range) age was 69 (57–84) years [9]. The median duration of treatment was 33.8 months in the D-VMP group versus 12.0 months in the VMP group. Mean baseline PRO domain scores were comparable between treatment groups for both the EORTC QLQ-C30 and EQ-5D-5L (Table 1).

**Table 1:** Baseline EORTC QLQ-C30 and EQ-5D-5L scores (ITT population)

	D-VMP (*n* = 146)	VMP (*n* = 74)
EORTC QLQ-C30 scores^a^		
GHS score	58.0 (22.0)	56.6 (23.1)
Physical functioning	73.7 (23.4)	71.6 (22.2)
Role functioning	71.4 (28.1)	70.1 (29.5)
Emotional functioning	79.7 (21.4)	79.5 (20.2)
Cognitive functioning	83.1 (19.3)	80.2 (17.6)
Social functioning	70.3 (26.6)	73.0 (25.8)
Pain	31.3 (28.2)	35.6 (28.8)
Fatigue	32.5 (21.8)	27.3 (17.3)
Nausea and vomiting	4.5 (11.3)	5.6 (10.4)
Dyspnea	15.5 (19.6)	15.3 (20.8)
Loss of appetite	16.4 (24.8)	16.2 (21.5)
Insomnia	19.9 (25.2)	18.9 (20.7)
Constipation	16.2 (22.2)	17.6 (23.6)
Diarrhea	5.0 (12.6)	5.4 (12.4)
Financial difficulties	32.7 (30.4)	32.0 (31.4)
EQ-5D-5L scores^b^		
VAS	70.2 (18.1)	67.9 (17.4)
Utility score	0.65 (0.28)	0.65 (0.28)

Note: Data are expressed as mean (SD)

EORTC QLQ-C30, European Organisation for Research and Treatment of Cancer Quality of Life Questionnaire Core 30-item; EQ-5D-5L, EuroQol 5-dimensional descriptive system; ITT, intent-to-treat; D-VMP, daratumumab plus bortezomib/melphalan/prednisone; VMP, bortezomib/melphalan/prednisone; GHS, Global Health Status; VAS, visual analog scale; SD, standard deviation

^a^Higher EORTC QLQ-C30 scores represent greater GHS, better functioning, and worse symptoms

^b^Higher EQ-5D-5L scores represent better health

At baseline, the compliance rate was 100% in the D-VMP and VMP groups for EORTC QLQ-C30 and EQ-5D-5L. Compliance rates remained relatively high by Month 12 in both groups (EORTC QLQ-C30, D-VMP = 82.6%, VMP = 67.4%; EQ-5D-5L, 82.6%, 67.4%), with rates remaining > 70% in the D-VMP group by Month 36 for both measures (78.9% for both EORTC QLQ-C30 and EQ-5D-5L; Online Resource 2).

### EORTC QLQ-C30 scores

**Fig. 1:**
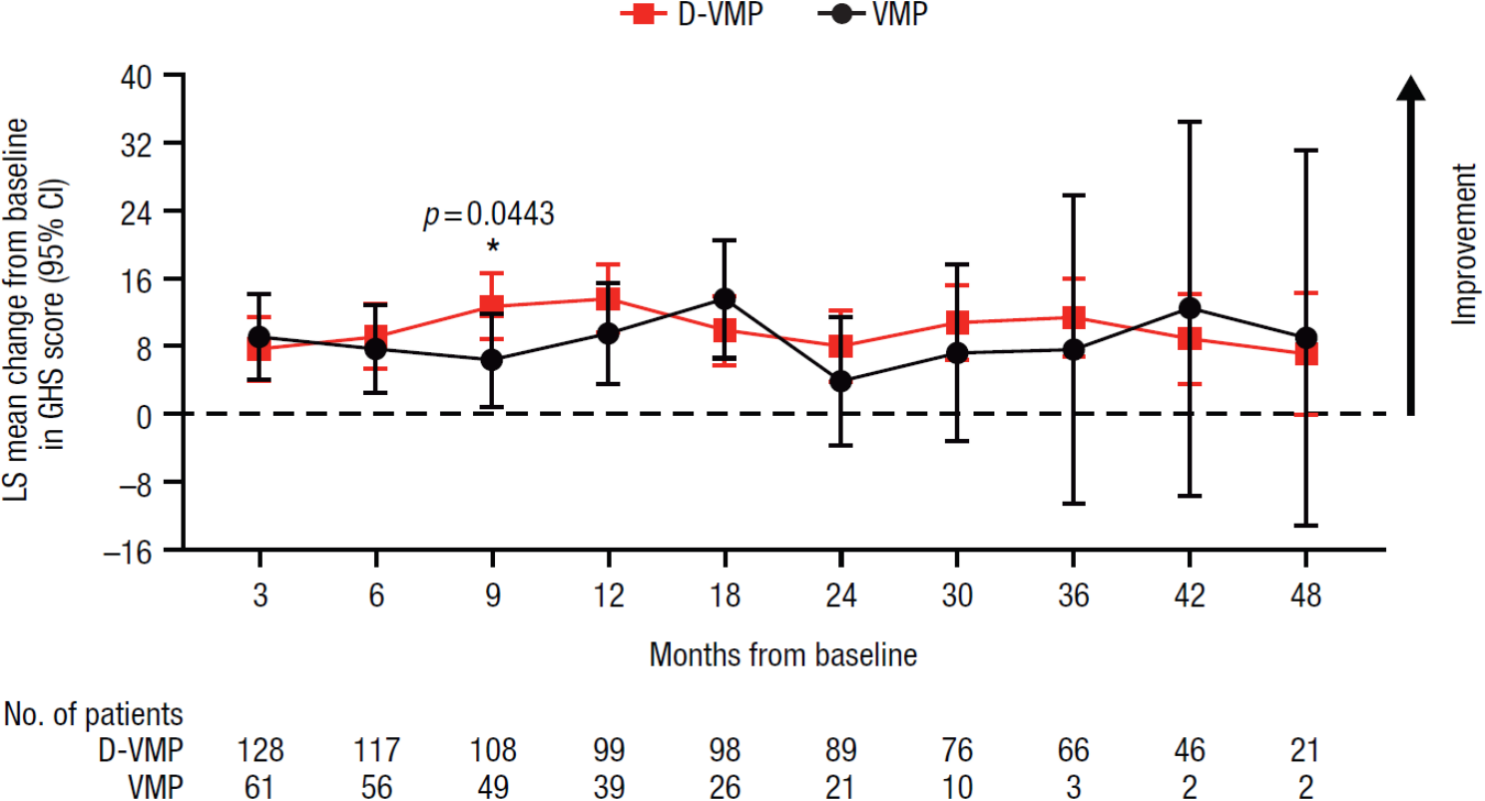
Change from baseline in EORTC QLQ-C30 GHS score. EORTC QLQ-C30, European Organisation for Research and Treatment of Cancer Quality of Life Questionnaire Core 30-item; GHS, Global Health Status; LS, least squares; CI, confidence interval; D-VMP, daratumumab plus bortezomib/melphalan/prednisone; VMP, bortezomib/melphalan/prednisone; ISS, International Staging System. LS means are derived based on the mixed-effects model with repeated measures, in which the dependent variable is change from baseline in score, and independent variables are baseline, visit, treatment, visit by treatment interaction, and randomization stratification factors—ISS staging (I, II, III) and age (< 75 years vs. ≥ 75 years)—as fixed effects and individual patient as random effect

A statistically significant improvement in GHS score from baseline to 9 months was observed in favor of D-VMP, with an LS mean change from baseline of 12.7 (95% CI, 8.8–16.6) in the D-VMP group and 6.4 (95% CI, 0.9–11.8) in the VMP group (difference, 6.3 [*p* = 0.0443]) (Fig. 1). No other significant differences in LS mean changes from baseline between treatment groups were observed at any other study time point for GHS.

**Fig. 2:**
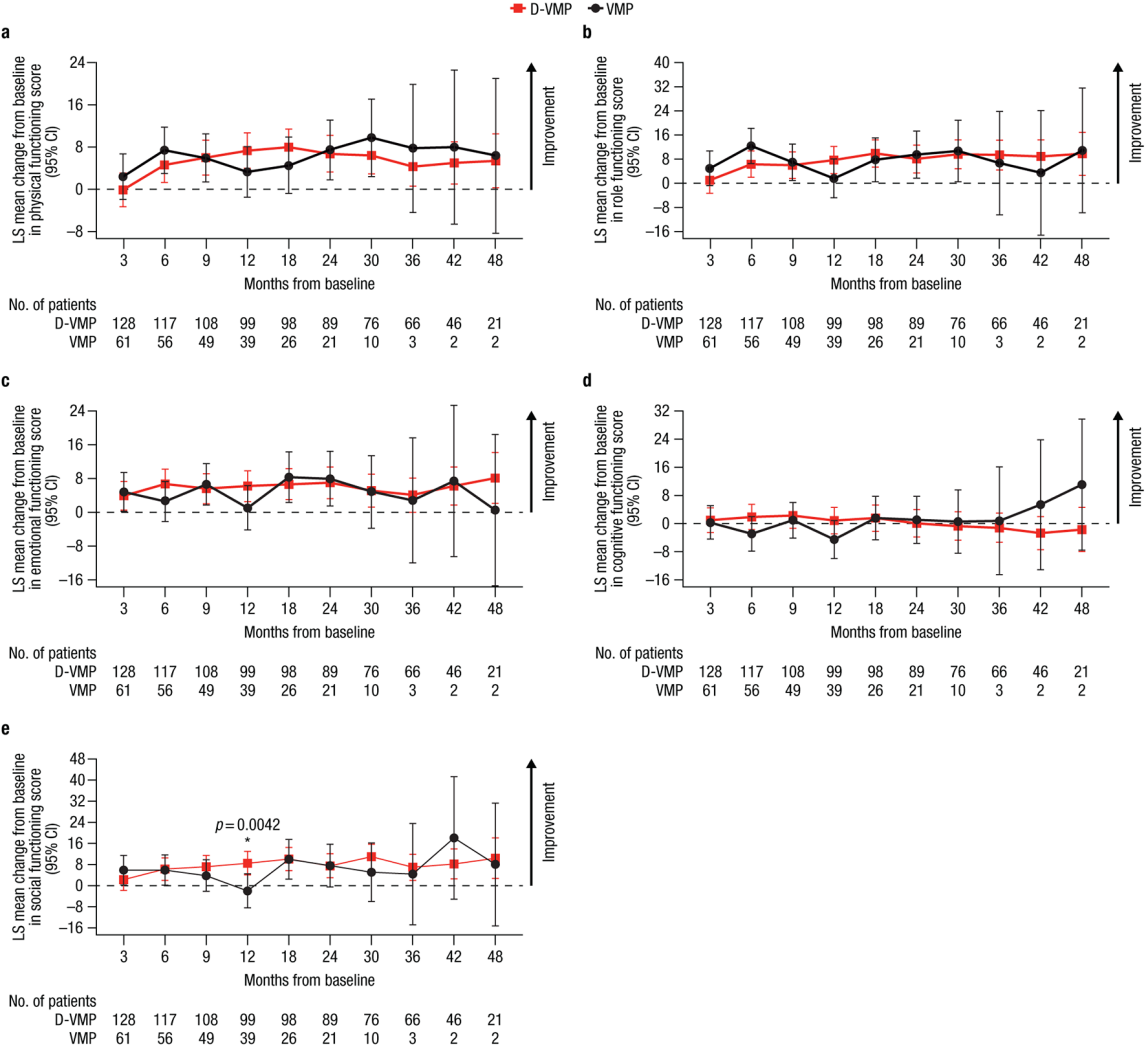
Change from baseline in EORTC QLQ-C30 functional domain scores for **a**) physical functioning, **b**) role functioning, **c**) emotional functioning, **d**) cognitive functioning, and **e**) social functioning. EORTC QLQ-C30, European Organisation for Research and Treatment of Cancer Quality of Life Questionnaire Core 30-item; LS, least squares; CI, confidence interval; D-VMP, daratumumab plus bortezomib/melphalan/prednisone; VMP, bortezomib/melphalan/prednisone; ISS, International Staging System. LS means are derived based on the mixed-effects model with repeated measures, in which the dependent variable is change from baseline in score, and independent variables are baseline, visit, treatment, visit by treatment interaction, and randomization stratification factors—ISS staging (I, II, III) and age (< 75 years vs. ≥ 75 years)—as fixed effects and individual patient as random effect

For EORTC QLQ-C30 functional domains, LS mean changes from baseline were generally comparable between treatment groups throughout the study (Fig. 2). Improvements from baseline in physical, role, emotional, and social functioning scores were observed across both treatment groups. A statistically significant improvement in social functioning from baseline to 12 months was observed in favor of D-VMP, with an LS mean change from baseline of 8.5 (95% CI, 4.1–13.0) in the D-VMP group versus −2.0 (95% CI, −8.4–4.5) in the VMP group (difference, 10.5 [*p* = 0.0042]). A decline in cognitive functioning from baseline was observed in both treatment groups, with a decline observed from 30 months onwards in the D-VMP group and at Months 6 and 12 in the VMP group.

**Fig. 3:**
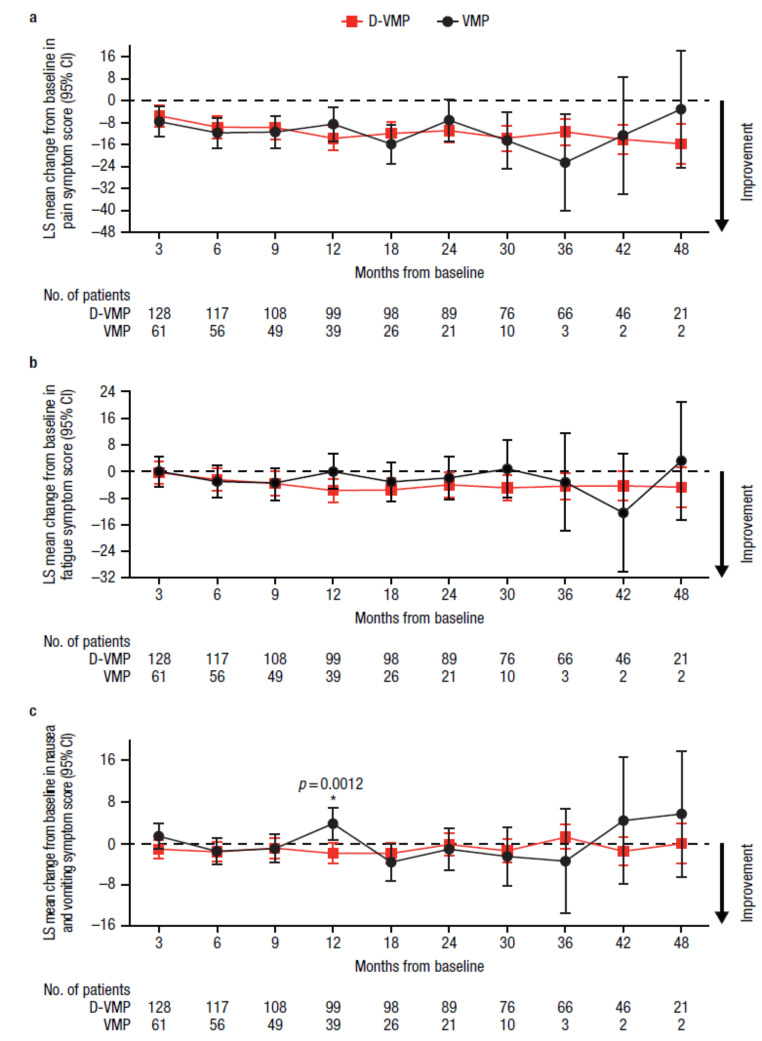
Change from baseline in EORTC QLQ-C30 symptom domain scores for **a**) pain, **b**) fatigue, and **c**) nausea and vomiting. EORTC QLQ-C30, European Organisation for Research and Treatment of Cancer Quality of Life Questionnaire Core 30-item; LS, least squares; CI, confidence interval; D-VMP, daratumumab plus bortezomib/melphalan/prednisone; VMP, bortezomib/melphalan/prednisone; ISS, International Staging System. LS means are derived based on the mixed-effects model with repeated measures, in which the dependent variable is change from baseline in score, and independent variables are baseline, visit, treatment, visit by treatment interaction, and randomization stratification factors—ISS staging (I, II, III) and age (< 75 years vs. ≥ 75 years)—as fixed effects and individual patient as random effect

Similarly, for EORTC QLQ-C30 symptom domains, LS mean changes from baseline were generally comparable between treatment groups throughout the study (Fig. 3). Improvements from baseline in fatigue were observed across all time points with D-VMP treatment, whereas fatigue worsened at 3 months with VMP treatment. Improvements from baseline in pain symptoms were observed across all time points for both D-VMP and VMP groups. A statistically significant improvement in nausea and vomiting symptoms from baseline to 12 months was observed in favor of D-VMP, with an LS mean change from baseline of −1.9 (95% CI, −3.9–0.1) in the D-VMP group versus 3.8 (95% CI, 0.7–6.8) in the VMP group (difference, −5.7 [*p* = 0.0012]).

**Fig. 4:**
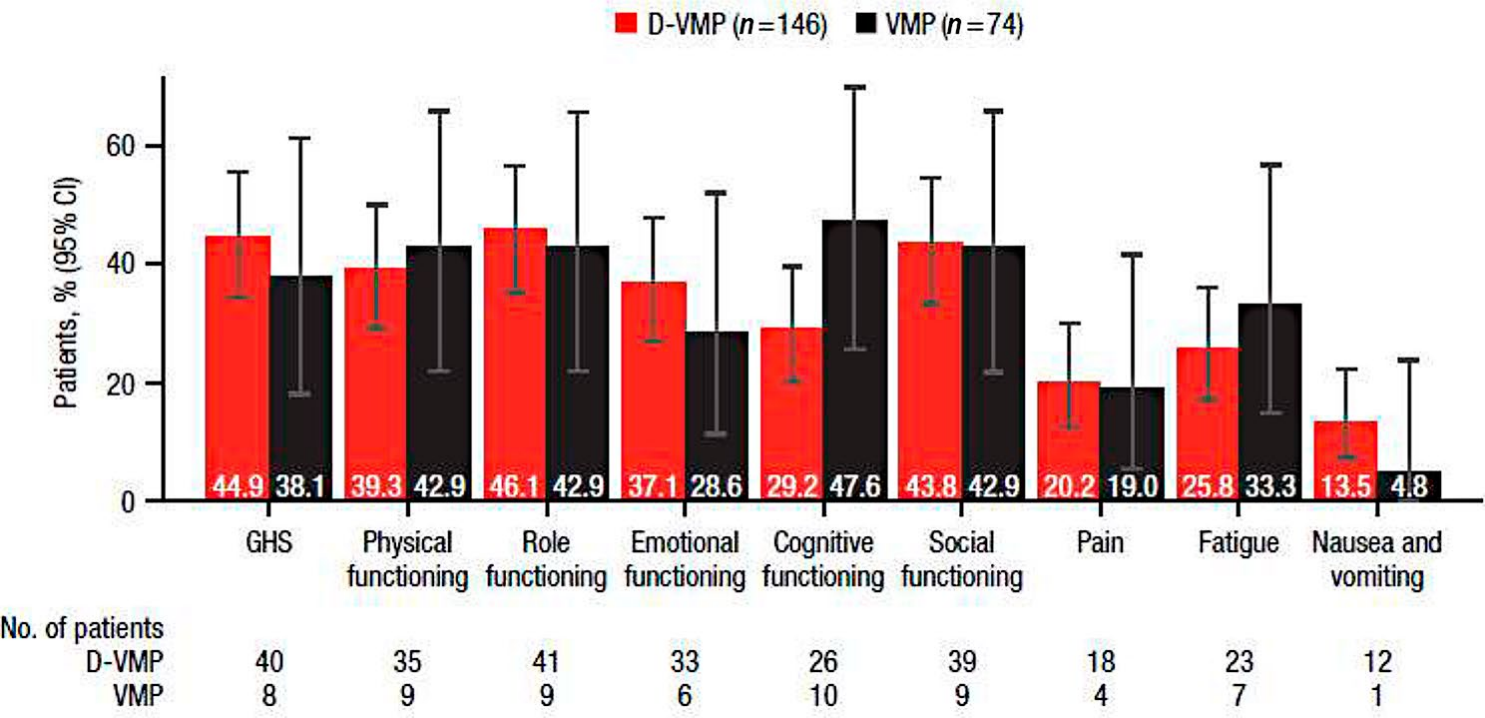
Percentage of patients reporting clinically meaningful improvement in EORTC QLQ-C30 GHS, functional, and symptom scales at 24 months. EORTC QLQ-C30, European Organisation for Research and Treatment of Cancer Quality of Life Questionnaire Core 30-item; GHS, Global Health Status; D-VMP, daratumumab plus bortezomib/melphalan/prednisone; VMP, bortezomib/melphalan/prednisone. Clinically meaningful improvement was defined as ≥ 10-point improvement from the baseline score. No. of patients is the number of patients with a minimally important difference in each treatment group

For EORTC QLQ-C30 functional and symptom domains, the percentages of patients with clinically meaningful changes in scores, defined as a ≥ 10-point change, at 24 months are shown in Fig. 4. Although not statistically significant, a numerically greater percentage of patients with clinically meaningful improvement was observed in favor of D-VMP compared with VMP across scores for GHS (44.9% vs. 38.1%), emotional functioning (37.1% vs. 28.6%), and nausea and vomiting (13.5% vs. 4.8%), while a numerically greater percentage was observed in favor of VMP in scores for cognitive functioning (29.2% vs. 47.6%) and fatigue (25.8% vs. 33.3%).

## EQ-5D-5L scores

**Fig. 5:**
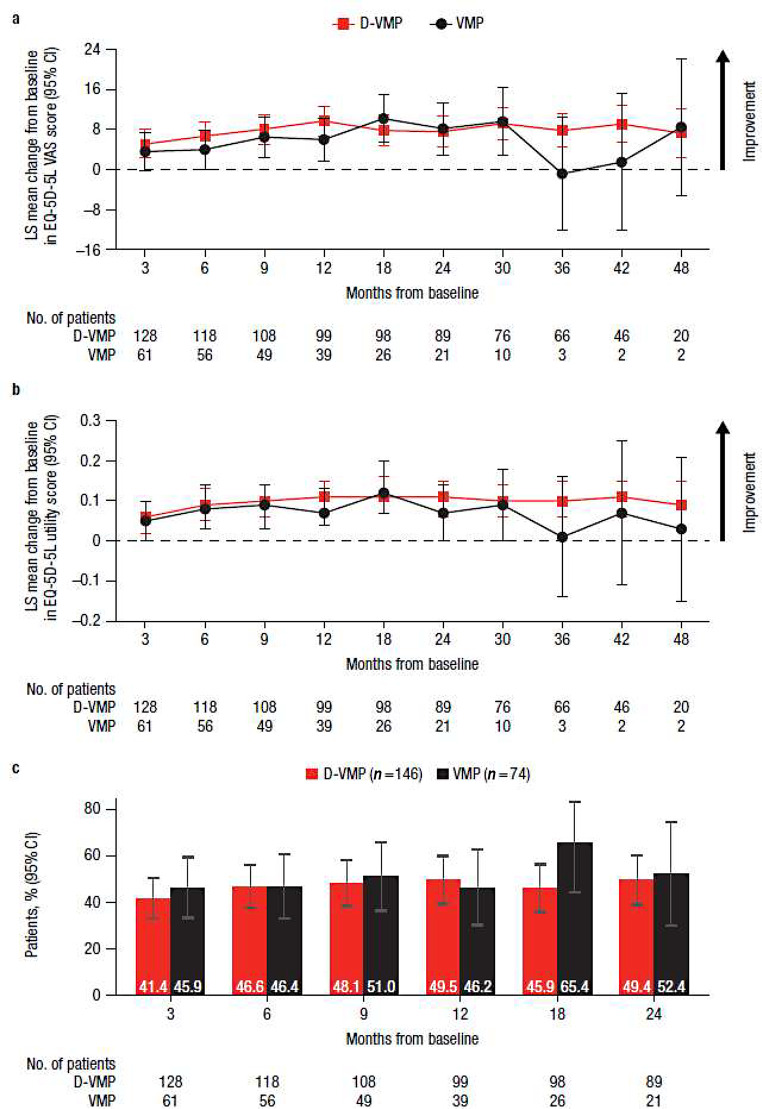
EQ-5D-5L results for **a**) change from baseline in VAS score, **b**) change from baseline in utility score, and **c**) the percentage of patients reporting clinically meaningful improvement in VAS score over 24 months. EQ-5D-5L, EuroQol 5-dimensional descriptive system; VAS, visual analog scale; LS, least squares; CI, confidence interval; D-VMP, daratumumab plus bortezomib/melphalan/prednisone; VMP, bortezomib/melphalan/prednisone; ISS, International Staging System. LS means are derived based on the mixed-effects model with repeated measures, in which the dependent variable is change from baseline in score, and independent variables are baseline, visit, treatment, visit by treatment interaction, and randomization stratification factors—ISS staging (I, II, III) and age (< 75 years vs. ≥ 75 years)—as fixed effects and individual patient as random effect. Clinically meaningful improvement was defined as ≥ 7-point improvement from the baseline score. No. of patients is the number of patients with VAS results in each treatment group at each time point

Both EQ-5D-5L VAS and utility scores improved from baseline in both treatment groups at the majority of study time points, with no statistically significant difference observed between groups (Fig. 5a and b). While the magnitude of improvement varied at different time points between D-VMP and VMP for EQ-5D-5L VAS, a greater numerical benefit in favor of D-VMP was seen across most time points in utility scores. For EQ-5D-5L VAS scores, comparable percentages of patients with clinically meaningful changes in scores, defined as a ≥ 7-point change, were generally observed for the D-VMP and VMP groups through 24 months (Fig. 5c).

## Discussion

Given the chronic nature of MM, understanding the impact of treatments on patient HRQoL is important for informing treatment decisions and complementing other clinical endpoints. This is particularly important for patients who are ineligible for transplant, as this group is commonly older, frail, and presenting with comorbidities. The importance of and use of PROs in clinical trials have steadily increased over time, with PRO data potentially assisting clinicians and patients during the treatment selection process in routine, real-world clinical practice [20]. Furthermore, PRO data may play a central role in ensuring a patient’s voice is incorporated into the drug development process, which is an emphasis of the “Guidance for Applying Patient-reported Outcomes (PROs) in Clinical Research for Drug Development (Interim)” PRO guidelines, published by China’s Center for Drug Evaluation in December 2021 [21, 22].

To our knowledge, this is one of the first studies to look at the impact of daratumumab-based therapies on PROs in Asian patients with transplant-ineligible NDMM. PRO data from this present analysis indicated that, in general, patients in both the D-VMP and VMP groups who remained in the study maintained their HRQoL during treatment. While improvements were generally comparable throughout the study, significantly greater improvements were observed in GHS at 9 months and social functioning and nausea and vomiting symptoms at 12 months following D-VMP versus VMP treatment. Improvements in general health status (EQ-5D-5L measures) were also sustained through the duration of the study, with less fluctuation in scores observed with D-VMP versus VMP. Furthermore, clinically meaningful improvements in EORTC QLQ-C30 and EQ-5D-5L scores were observed in both treatment groups over 24 months. The percentage of patients with clinically meaningful differences was numerically greater in the D-VMP group for GHS, emotional functioning, and the nausea and vomiting symptom score, but was numerically greater in the VMP group for cognitive functioning and the fatigue symptom score. Together, these findings suggest the addition of daratumumab to VMP does not negatively impact HRQoL and may provide a benefit in overall HRQoL in patients with MM.

While cross-trial comparisons should be interpreted with caution, the improvements in HRQoL in D-VMP- and VMP-treated, transplant-ineligible patients with NDMM in the current analysis are comparable to those seen in the global phase 3 ALCYONE study [13]. In ALCYONE, improvements from baseline were observed across both D-VMP and VMP treatment groups for EORTC QLQ-C30 GHS and most functional and symptom scales, and for the EQ-5D-5L VAS. Similar to that reported here, a significant improvement in GHS was observed with D-VMP versus VMP; however, this occurred earlier in ALCYONE (*p* = 0.0240 at 3 months) compared with OCTANS (*p* = 0.0443 at 9 months). Clinically meaningful improvements from baseline were also generally observed across both treatment groups for EORTC QLQ-C30 scales and EQ-5D-5L VAS in ALCYONE, which is consistent with the current study. In both the current study and ALCYONE, a decline in cognitive functioning was observed at certain time points in both treatment groups. A similar observation was also made in the global phase 3 MAIA study of daratumumab combined with standard-of-care lenalidomide and dexamethasone (Rd) doublet versus Rd alone, in which a reduction in cognitive functioning was reported for both treatment groups at Cycles 6 and 12 [12]. While the magnitude of decline was not clinically meaningful in OCTANS or ALCYONE, this may warrant further investigation given that cancer-related cognitive impairment, while present, is understudied in MM [23].

The benefit of daratumumab-based regimens versus their respective standard-of-care regimens with respect to PROs and HRQoL has also been observed in other clinical trials. In the global phase 3 MAIA study, the addition of daratumumab to Rd was associated with faster and sustained clinically meaningful improvements in GHS and pain versus Rd alone in transplant-ineligible patients with NDMM [12]. Furthermore, in the phase 2 GRIFFIN study and phase 3 CASSIOPEIA study, the addition of daratumumab to lenalidomide, bortezomib, and dexamethasone (RVd) and to bortezomib, thalidomide, and dexamethasone (VTd), respectively, resulted in greater improvements in HRQoL domains than RVd and VTd regimens alone [14, 24].

In a real-world study of 330 patients with MM, an increase in reported pain severity was associated with a significant decrease in overall HRQoL, thus highlighting the importance of pain management in this patient population [25]. Notably, in the current study, sustained and clinically meaningful improvements in pain were observed across both D-VMP and VMP treatment groups throughout the entire study. Moreover, improvements in pain in the D-VMP group were accompanied by steady, prolonged improvements in GHS and VAS. In addition to pain, fatigue is a common symptom of MM, which can often be exacerbated by treatments such as chemotherapy. In a comprehensive meta-analysis of 34 MM studies, fatigue was among one of the most prevalent symptoms, reported in 98.8% of patients [26]. Given that fatigue can frequently affect daily activities, concentration, and motivation and interfere with work and social life, the management of this prevalent symptom is crucial. Notably, in the current study, D-VMP resulted in greater sustained, stable improvements in fatigue from baseline throughout the study versus VMP.

Considering the high symptom burden faced by many patients with NDMM, particularly those who are transplant-ineligible due to age or comorbidities, the addition of any MM therapy to already increasingly complex novel-agent–based regimens may raise concerns for treatment tolerance and toxicity [27]. In the primary analysis of OCTANS, the addition of daratumumab to VMP led to significant improvements in response rates, MRD-negativity rates, and progression-free survival, with a manageable safety profile, in transplant-ineligible Asian patients with NDMM [9]. With an extended median follow-up of > 3 years in OCTANS, D-VMP continued to demonstrate a clinical benefit versus VMP alone, with no new safety concerns [10]. Therefore, results from the current PRO analysis, in addition to the previously reported clinical efficacy and safety data, further support the addition of daratumumab to VMP in transplant-ineligible patients with NDMM, without compromise to HRQoL.

A limitation of the current analysis is the overall sample size. As the study progressed, the sample size in each treatment group at each time point decreased, thus limiting the conclusions that can be drawn from the data at later time points. In addition, due to the 2:1 randomization per the OCTANS study design, the number of patients randomized to the D-VMP group was greater than the number of patients randomized to the VMP group, and while patients in the D-VMP group continued daratumumab until disease progression, unacceptable toxicity, or study end, patients in the VMP group received VMP for a fixed duration of up to 9 cycles. Therefore, PRO data were collected from fewer patients and at fewer time points in the VMP group compared with the D-VMP group, thereby limiting comparisons between treatment groups after 24 months. Furthermore, patients in the D-VMP group also received pre-infusion medications with dexamethasone, paracetamol, and antihistamine before each daratumumab infusion; these additional medications in the D-VMP group specifically may have consequently impacted PRO responses.

In conclusion, this is the first study to demonstrate the improvement in HRQoL with either D-VMP or VMP treatment in Asian transplant-ineligible patients with NDMM. Improvements in HRQoL scores and a notable percentage of patients achieving clinically meaningful improvements from baseline were noted across most functional and symptom domains, and a sustained benefit in general health status was also observed throughout the study. The addition of daratumumab to VMP, however, led to improvements in GHS and life-impacting functional and symptoms scores, such as social functioning and nausea and vomiting, versus VMP alone. These HRQoL benefits are consistent with the efficacy benefits observed with D-VMP versus VMP in the OCTANS study, and further corroborate the HRQoL benefits observed with D-VMP versus VMP in ALCYONE. Overall, these results emphasize the benefit of therapy on the day-to-day lives of patients with MM and provide additional support for the use of daratumumab-based regimens such as D-VMP in transplant-ineligible patients with NDMM.

## Electronic supplementary material

Below is the link to the electronic supplementary material.

Supplementary Material 1

## Data Availability

The data sharing policy of Johnson & Johnson is available at https://innovativemedicine.jnj.com/our-innovation/clinical-trials/transparency. As noted on this site, requests for access to the study data can be submitted through the Yale Open Data Access (YODA) Project site at http://yoda.yale.edu.
