# Supplementary material for: Health-related quality of life with daratumumab, bortezomib, melphalan, and prednisone versus bortezomib, melphalan, and prednisone alone in transplant-ineligible patients with newly diagnosed multiple myeloma: analysis of the phase 3 OCTANS study
Source: Ann Hematol. 2025 May 23;104(5):2765–76. doi: 10.1007/s00277-025-06303-3 (PMC12141397; doi:10.1007/s00277-025-06303-3)
Supplement: Supplementary file 1 — Supplementary Material 1 [file 277_2025_6303_MOESM1_ESM.pdf]

Supplementary Appendix

Health-related Quality of Life With Daratumumab, Bortezomib, Melphalan, and Prednisone Versus Bortezomib, Melphalan, and Prednisone Alone in Transplant-ineligible Patients With Newly Diagnosed Multiple Myeloma: Analysis of the Phase 3 OCTANS Study

Annals of Hematology

Weijun Fu, Soo-Mee Bang, Honghui Huang, Kihyun Kim, Wei Li, Gang An, Je-Jung Lee, Zhen Cai, Jie Jin, Yafei Wang, Chor Sang Chim, Robin Carson, Rui Liu, Man Zhao, Xi Chen, Canchan Cui, Jian Hou, Jianxiang Wang

Corresponding authors:

Prof. Jian Hou

Renji Hospital, Shanghai Jiao Tong University School of Medicine, Shanghai, China

Email: houjian@medmail.com.cn

Prof. Jianxiang Wang

Chinese Academy of Medical Sciences & Peking Union Medical College, Tianjin, China

Email: wangjx@ihcams.ac.cn

Online Resource 1. Demographic and baseline disease characteristics (ITT population)

**Characteristic**	**D-VMP (n=146)**	**VMP (n=74)**
Age		
Median (range), years	69 (58-81)	69 (57-84)
Distribution, n (%)		
<65 years	3 (2.1)	1 (1.4)
65 to <75 years	120 (82.2)	63 (85.1)
≥75 years	23 (15.8)	10 (13.5)
Sex, n (%)		
Male	85 (58.2)	46 (62.2)
Female	61 (41.8)	28 (37.8)
ECOG PS score, n (%)		
0	50 (34.2)	21 (28.4)
1	71 (48.6)	40 (54.1)
2	25 (17.1)	13 (17.6)
ISS staging, n (%)[a]		
I	37 (25.3)	19 (25.7)
II	68 (46.6)	32 (43.2)
III	41 (28.1)	23 (31.1)
Time from MM diagnosis to randomization		
Median (range), months	0.7 (0.1-14.6)	0.6 (0.1-2.1)
Cytogenetic risk[b]		
n	145	74
Standard risk, n (%)	117 (80.7)	54 (73.0)
High risk, n (%)	28 (19.3)	20 (27.0)
del(17p)	15 (10.3)	9 (12.2)
t(4;14)	15 (10.3)	12 (16.2)
t(14;16)	7 (4.8)	2 (2.7)

ITT, intent-to-treat; D-VMP, daratumumab plus bortezomib/melphalan/prednisone; VMP, bortezomib/melphalan/prednisone; ECOG PS, Eastern Cooperative Oncology Group performance status; ISS, International Staging System; MM, multiple myeloma; FISH, fluorescence in situ hybridization

[a]ISS staging is derived based on the combination of serum β_2-microglobulin and albumin

[b]Cytogenetic risk was based on local FISH or karyotype testing. Standard cytogenetic risk was defined as none of the following high-risk cytogenetic abnormalities: del(17p), t(4;14), or t(14;16). High cytogenetic risk was defined as ≥1 of the following high-risk cytogenetic abnormalities: del(17p), t(4;14), or t(14;16)

Online Resource 2. Patient-reported outcome instrument compliance rates during treatment (ITT population)

	**D-VMP (n=146)**			**VMP[a] (n=74)**		
	Expected	Received	Compliance (%)[b]	Expected	Received	Compliance (%)[b]
**EORTC QLQ-C30**						
Baseline	146	146	100	74	74	100
Month 3	133	125	94.0	66	58	87.9
Month 6	129	112	86.8	60	51	85.0
Month 9	119	103	86.6	53	43	81.1
Month 12	115	95	82.6	46	31	67.4
Month 18	110	89	80.9	3	3	100
Month 24	98	79	80.6	-	-	-
Month 30	85	66	77.6	-	-	-
Month 36	76	60	78.9	-	-	-
Month 42	58	39	67.2	-	-	-
Month 48	22	15	68.2	-	-	-
Month 54	12	6	50.0	-	-	-
Month 60	5	2	40.0	-	-	-
**EQ-5D-5L**						
Baseline	146	146	100	74	74	100
Month 3	133	125	94.0	66	58	87.9
Month 6	129	113	87.6	60	51	85.0
Month 9	119	103	86.6	53	43	81.1
Month 12	115	95	82.6	46	31	67.4
Month 18	110	89	80.9	3	3	100
Month 24	98	79	80.6	-	-	-
Month 30	85	66	77.6	-	-	-
Month 36	76	60	78.9	-	-	-
Month 42	58	39	67.2	-	-	-
Month 48	22	15	68.2	-	-	-
Month 54	12	6	50.0	-	-	-
Month 60	5	2	40.0	-	-	-

ITT, intent-to-treat; D-VMP, daratumumab plus bortezomib/melphalan/prednisone; VMP, bortezomib/melphalan/prednisone; EORTC QLQ-C30, European Organization for Research and Treatment of Cancer Quality of Life Questionnaire Core 30-item; EQ-5D-5L, EuroQol 5-dimensional descriptive system; PRO, patient-reported outcome

[a]Only PRO assessments during the treatment phase were counted towards compliance. For the VMP group, treatment was given for a maximum of 9 cycles; thus, no additional compliance data were collected for the VMP group following Month 18

[b]Compliance was defined as the number of forms received as a percentage of the number of forms expected. Forms were expected from all patients who were on study treatment by certain month cut-off. Percentages calculated with the number of expected forms in each group as the denominator
